# Barriers and facilitators to provide multidisciplinary care for breast cancer patients in five Latin American countries: A descriptive-interpretative qualitative study

**DOI:** 10.1016/j.lana.2022.100254

**Published:** 2022-04-07

**Authors:** Carlos Barrios, Guillermo Sánchez-Vanegas, Cynthia Villarreal-Garza, Andrés Ossa, Milton A. Lombana, Angélica Monterrosa-Blanco, Ana S. Ferrigno, Carlos Alberto Castro

**Affiliations:** a Oncoclinicas Group, Oncology Research Center, Hospital São Lucas, PUCRS Latin-American Cooperative Oncology Group (LACOG), Rio Grande do Sul, RS, Brazil; b Soluciones Integrales Para la Investigación y la Educación en Salud – SIIES Consultores, Cr 45ª #106ª-20, Bogotá, Cundinamarca, Colombia; c Breast Cancer Center, Hospital Zambrano Hellion TecSalud, Tecnologico de Monterrey, San Pedro Garza García, Nuevo León, México; d Coordinator Breast Cancer Department Hospital General de Medellín, Medellín, Antioquia, Colombia; e Scientific Medical Head at Comprehensive Cancer Center, Clinica de Occidente, Cali, Valle del Cauca, Colombia; f Fundación Universtiaria de Ciencias de la Salud-FUCS, Bogotá, Colombia.

**Keywords:** Breast cancer, Latin America, Multidisciplinary care, Interdisciplinary team, Patient management

## Abstract

**Background:**

Multidisciplinary care (MDC) remains a cornerstone for breast cancer management as it is associated with improved quality of care and patient outcomes. However, the adoption of MDC practice is heterogeneous and has been poorly explored in Latin America. The objective was to describe barriers and possible facilitators for providing MDC to breast cancer patients in five Latin American countries.

**Methods:**

A panel of experts with an active clinical practice in Bolivia, Colombia, Ecuador, Mexico, and Uruguay was convened to identify barriers and facilitators to MDC. This study is a qualitative synthesis of a structured discussion regarding the state of MDC in the setting of breast cancer.

**Findings:**

Experts recognized that most oncology practices in Latin America do not apply a multidisciplinary approach for breast cancer patients. Predominant barriers for MDC are fragmentation of health services, being understaffed, inadequate infrastructure, and geographic disparities. Access to MDC varies widely in the region, with significant heterogeneity documented within countries. MDC practice was described as being more common in the private sector in Ecuador and Uruguay, while it is more widely implemented in public institutions of Colombia and Bolivia.

**Interpretation:**

Establishing quality MDC remains a challenge for oncology practices in Latin America. Addressing regional issues and identifying specific local needs is warranted to encourage the adoption of an effective multidisciplinary approach and, consequently, improve clinical outcomes. Active involvement of all stakeholders is required to build locally solutions and should involve institutions, health professionals, and patients.

**Funding:**

Research was funded by Productos Roche S.A.

**Research in context**

**Evidence before this study**

For approximately 26 years, specialized cancer organizations have promoted the concept of multidisciplinary care as a critical element in cancer patient care, which arose from the need to provide comprehensive care, make informed decisions, unify clinical practice and improve health outcomes. Worldwide, countries like the United Kingdom and China have the most experience and track record implementing multidisciplinary care. This has made it possible to demonstrate with accurate figures that multidisciplinary care can increase disease-free survival and overall survival compared to patients who do not undergo comprehensive and multidisciplinary management. On the contrary, Latin American healthcare providers indicate the absence of multidisciplinary meetings at their worksites in several available surveys. Therefore, the scientific literature on this practice in Latin America is scarce.

**Added value of this study**

Breast cancer experts from five Latin American countries shared their experience about how the process of multidisciplinary care has been implemented in their countries. The estimated percentage of breast cancer patients who have access to multidisciplinary care differs between countries and public and private care sectors. None of the included countries have systematic and formal measurements on the impact of multidisciplinary care in patients with breast cancer. In addition, barriers to multidisciplinary care were identified from three different perspectives: the health system, health professionals, and patients. Likewise, to improve multidisciplinary care, some strategies are proposed, and those that have been effective are highlighted, such as virtual participation of international experts in regional tumor boards, periodic meetings with insurers to audit treatment decisions and results, documentation of multidisciplinary care decisions in patients' medical records, among others.

**Implications of all evidence available**

Knowing the benefits of multidisciplinary care in terms of favorable clinical outcomes for the patient motivates its implementation and constant development. In order to achieve systematic and standardized medical management, it is also necessary to bear in mind which are the different barriers in the local context that hinder the possibility of offering multidisciplinary approach to breast cancer patients. Considering the barriers also contributes to the generation of solutions and strategies that involve patients and health professionals. In addition, through these findings, governmental and institutional entities and stakeholders from Latin American countries must be aware of the importance and need to adopt a multidisciplinary care of breast cancer.

Alt-text: Unlabelled box

## Introduction

Breast cancer is the most commonly diagnosed malignancy and the fifth leading cause of cancer mortality, with 2·3 million new cases and 685,000 deaths registered worldwide in 2020.^1^ Latin America represents a major public health and economic issue, and its burden is expected to increase due to the ongoing demographic transition.^2^^,^^3^ Of note, a high mortality-to-incidence ratio (MIR) is present throughout the region. According to GLOBOCAN 2020, age-standardized incidence and mortality rates were estimated at 56·4 cases and 14·0 deaths in South America and 39·5 cases and 10·4 deaths per 100,000 person-years in Central America (MIR of 0·25 and 0·26, respectively), which compares unfavorably to the 89·4 cases and 12·5 deaths per 100,000 person-years documented in North America (MIR 0·14).^1^ As breast cancer mortality in Latin America is expected to double within the next 20 years,^4^ the development and implementation of targeted strategies to improve patient outcomes are urgently needed. Promoting and advancing multidisciplinary care (MDC) is a promising strategy to improve breast cancer outcomes in the region.

The diagnosis, staging, and treatment of breast cancer require multiple healthcare providers with different areas of specialization. A multidisciplinary approach is recognized as the standard of care because it allows for enhanced communication between the different disciplines involved in patient management and permits the coordination of services to improve patient care.^5^ This concept arose from the need to provide comprehensive multimodality care, make evidence-based decisions, limit disparities, and unify practices in the era of personalized medicine.^6^ Because of its potential benefits, the adoption of MDC is endorsed by cancer organizations such as the American Society of Clinical Oncology (ASCO) and the European Society for Medical Oncology (ESMO) as an integral component of optimal patient care.^7^

MDC in the form of interdisciplinary meetings has proven to be beneficial in terms of decreasing time from diagnosis to treatment, promoting complete pre-operative staging, tailoring treatment recommendations to individual case characteristics, increasing patient satisfaction, and possibly achieving improved survival outcomes.^8^, ^9^, ^10^, ^11^, ^12^, ^13^, ^14^, ^15^ Additionally, physicians participating in tumor boards report that this strategy can increase team competence and improve collegiality.^16^^,^^17^ Despite its positive impact on patient care, multidisciplinary tumor boards (MTB) have been heterogeneously adopted in clinical practice.^18^^,^^19^ Multiple barriers have been identified for its implementation, including excessive administrative time, high cost, insufficient reimbursement, excessive caseload, inadequate attendance, and lack of leadership.^20^^,^^21^

In Latin America, data on the implementation and outcomes of MTB is scarce. In an international cross-sectional survey of healthcare providers participating in an ongoing clinical trial, only 6·7% of participants from countries of Latin America (i.e., Argentina, Brazil, Chile, and Peru) reported that multidisciplinary meetings were not available in their institution.^19^ However, in another survey distributed through social media, 14% of clinicians from Latin America reported that they did not have regular tumor boards in their centers.^22^ However, to our knowledge, no information is available regarding the challenges to implement MDC in the region or possible strategies to overcome them. The present study aims to describe the barriers and potential facilitators for MDC in the form of interdisciplinary meetings for breast cancer patients in five countries to better characterize the state of this strategy in Latin America.

## Methodology

### Design

A descriptive-interpretative qualitative research was carried out using MDC of breast cancer patients in Latin America as a case study and employing the expert panel technique.

### Participants

Two working groups were established for this study. The first, called the developer group (DG), included four specialists (three medical oncologists and a breast surgeon from Brazil, Colombia, and Mexico) and a team of experts in research methodology who were in charge of the study design, data collection, and synthesis of the findings. The second, called the expert panel (EP), comprised a group of physicians from Bolivia, Colombia, Ecuador, Mexico, and Uruguay that included eight medical oncologists, six breast cancer surgeons, seven pathologists, and five radiation oncologists. Participants' medical specialties and country of practice are detailed in Supplementary Material 1.

### Data collection

The study was divided into four phases. First, the DG determined the objectives and scope of the study. A set of guiding questions that would be shared with the EP was selected. Each of these questions was designed to assess the definition and implementation of MDC in their country, existing barriers for its use in the public and private health sectors, probable facilitators for its application, and the potential impact that it could have in terms of patient care. The final set of guiding questions is shown in Supplementary Material 2. In the second phase, the principal investigator presented the study protocol to the EP and instructed them to evaluate the use of MDC in their country. Consequently, the members of the EP from each country registered the prevailing regional consensus on the topics addressed in the guiding questions. Lastly, the findings were presented at a virtual plenary session with all members of the DG and EP. A spokesperson of each country was appointed and presented the local findings using a standardized format. At the end of each presentation, there was an allotted time for queries, comments, and discussion among participants.

### Information analysis

The research experts consolidated the findings presented at the plenary session. In some cases, it was necessary to contact the groups from each country to clarify or supplement their findings. The results presented below represent an analytical synthesis prepared by the DG based on the findings discussed at the plenary session.

### Role of the funding source

Productos Roche S.A funded the research group activities, including design, data collection, and analysis, but they did not intervene and stay sidelines of the carried-out activities.

## Results

### Definition of multidisciplinary care and proposed participating members

The experts of each country had slightly different definitions of MDC and ideas about the team members that should participate in this strategy (Supplementary Material 3). However, all countries agreed that MDC involved the discussion of healthcare providers with different areas of expertise. Furthermore, most countries recognized that the objective was to provide tailored management recommendations based on individual case characteristics.

The DG synthetized the definitions of each country and concluded that: *MDC comprises the active collaboration of a group of professionals from different specialties who, based on the best available scientific evidence and considering the opinion of all collaborators, plan an optimal diagnostic strategy and therapeutic sequence, ideally leading to the best possible clinical outcome for patients.*

**Figure 1:**
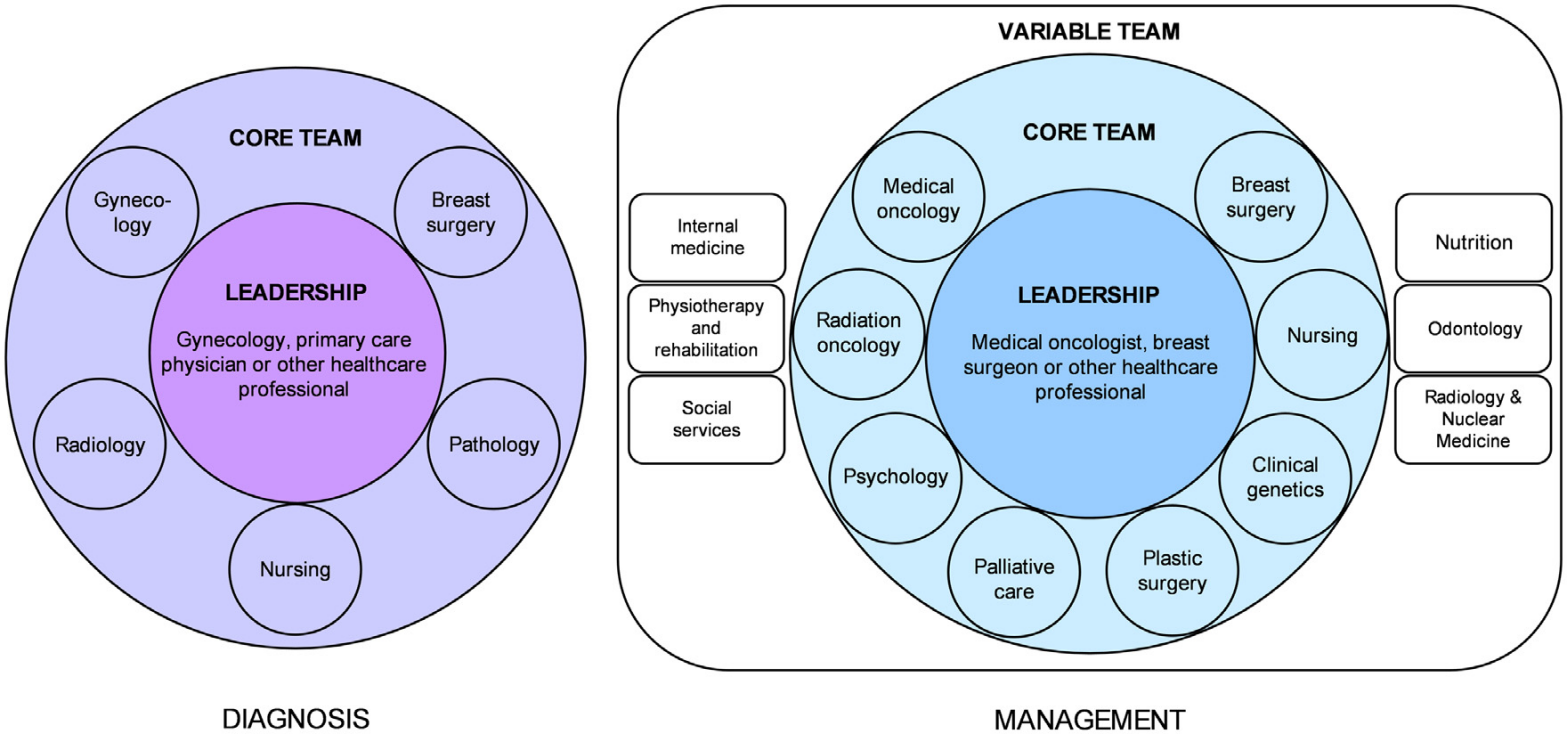
Proposed composition of MDC teams according to the management stage.

The EP concluded that MDC teams must include a variety of specialists to guarantee comprehensive care. However, the exact composition of the MDC team should be tailored according to patients' specific needs and stages of their oncologic management, as well as regional availability and resources. Based on the input from the EP, the optimal human elements required for MDC could be categorized as shown in Figure 1. The core team, composed of medical oncologists, breast surgeons, radiation oncologists, plastic surgeons, palliative care specialists, psychologists, nurses, and clinical geneticists, was identified as the group of professionals that should participate when providing MDC. In addition, a variable group was defined as healthcare providers with different areas of expertise who could supplement the discussion and be invited on a case-by-case basis.

While optimal MDC requires input from healthcare providers with a wide variety of areas of expertise, it was agreed that the participants of regular MTB should be determined according to available resources. The experts recognized the discussion of individual case characteristics among specialists in medical oncology, breast surgery, and radiation oncology as essential to developing tailored treatment plans, optimizing patient care, and possibly reducing healthcare costs. While the ideal method to undertake such discussions would be in the form of institutional tumor boards, it was recognized that at least informal discussions of optimal treatment strategies for each case (either in person, by phone call, or using teleconferencing) could be beneficial in terms of patient care. Ultimately, context-dependent strategies to promote a multidisciplinary approach when developing diagnostic and management strategies are needed to increase MDC uptake.

### Access to MDC in the public and private context

**Figure 2:**
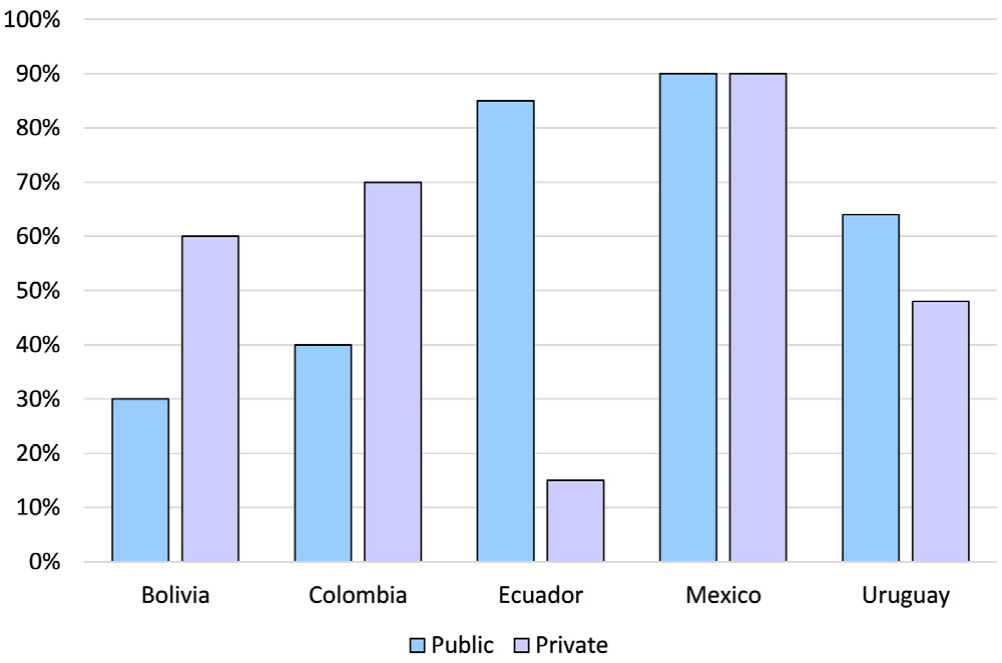
Estimated access to MDC according to the type of healthcare system and country [also see Supplementary Material 4].

The members of the EP recognized that no formal data exists at a national level to evaluate access to MDC in their respective countries. Hence, the estimates they could provide on the uptake of this strategy in breast cancer are based on anecdotal information. In their experience, access to MDC in the region is heterogeneous, with substantial differences between private and public health institutions and among individual centers. In the private sector, the percentage of cases managed through MDC according to the EP varies from 15% in Ecuador to 90% in Mexico, while it ranged from 30% in Bolivia to 90% in Mexico in the context of public healthcare (Figure 2, Supplementary Material 4). Notably, EP from Montevideo, Uruguay, and select academic centers in Mexico declared that patients always have access to MDC. In addition, the panelists agreed that urban centers had a higher tendency to provide MDC than those in rural settings.

### Barriers to MDC and proposed strategies

**Figure 3:**
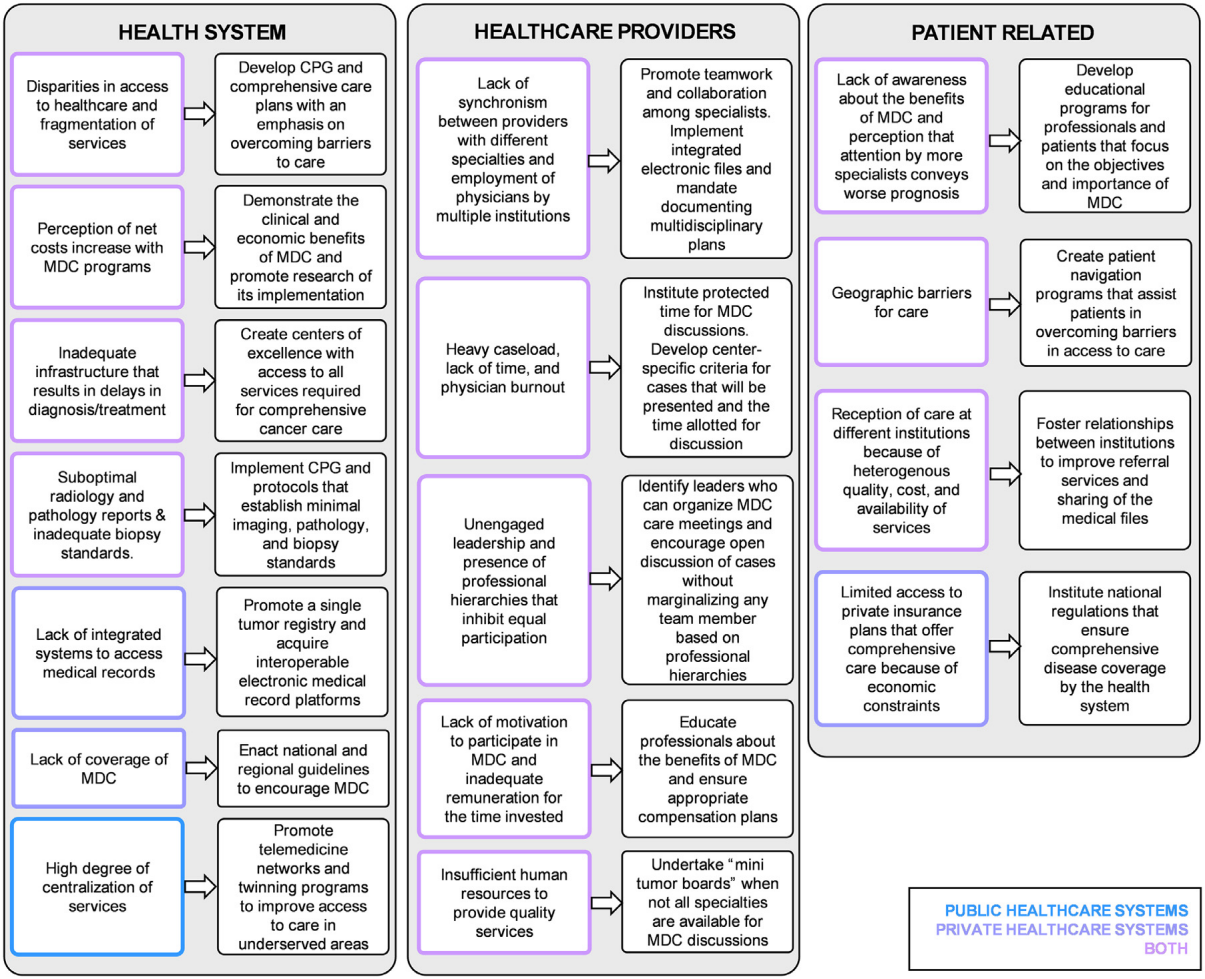
Barriers identified for MDC and proposed facilitators to overcome them.

In Figure 3*,* the barriers to MDC identified in the public and private sector and potential strategies to overcome them are presented and grouped into three domains according to their origin: healthcare system, healthcare workers, and patient related. Of note, all challenges and possible strategies provided by the EP should be considered in a resource-dependent scenario. No single tactic to promote MDC would be appropriate or feasible in all regions to promote MDC in Latin America.

The barriers that hinder MDC at a system-level cited by the EP were similar in public and private healthcare institutions. Nonetheless, the panel recognized that one of the most detrimental barriers for MDC in the public sector was economic constraints due to coverage limitations, the type of hiring of the insurer, and the hiring of specialists.

Centralization of services was cited as the most significant challenge in private institutions because it requires that patients in remote areas move to main centers to access professionals with a high level of specialization. In terms of barriers pertaining to healthcare professionals, excessive workload, and difficulties to reach consensus among specialists were cited as the predominant barriers for MDC. Lastly, the most relevant patient-specific obstacle for receiving MDC cited was lack of knowledge about the benefits of MDC.

**Figure 4:**
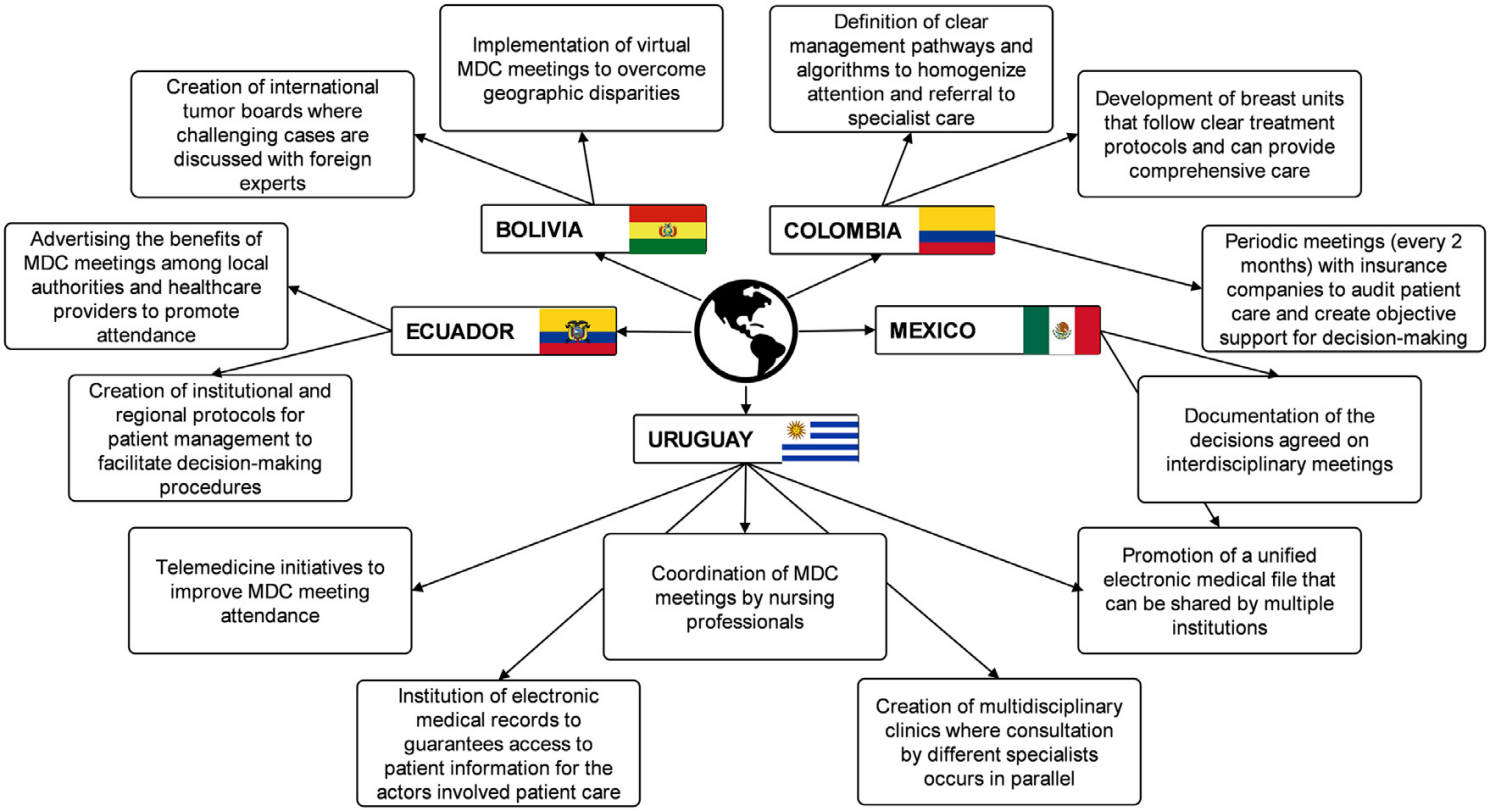
Strategies implemented in five Latin American countries to promote MDC.

Figure 4 highlights the strategies that have been successful in improving MDC access and implementation in the five Latin American countries, according to the EP. In Bolivia, the virtual participation of international experts in regional tumor boards has facilitated MDC by overcoming the shortage of cancer specialists in underserved areas. In Colombia, regular meetings with insurers to audit treatment decisions and outcomes have been useful for promoting comprehensive patient care. In Ecuador, participation in multidisciplinary sessions has been facilitated by disseminating its importance and objectives by health institutions among local healthcare providers and authorities. In Mexico, documentation of multidisciplinary care decisions in the medical files of patients has promoted their adherence. In Uruguay, coordination of regular meetings by nursing professionals has brought positive results.

### Measurements of the impact of multidisciplinary care

All participants agreed on the importance of measuring the impact of implementing multidisciplinary breast cancer care. However, a formal and systematic evaluation of MDC has not been performed in none of the countries. The EP specified that not having established guidelines and objective metrics to follow contributes to the lack of implementation of MDC.

Panelists from Bolivia, Ecuador, and Uruguay declared that they did not have any data on MDC at their institutions. However, it was highlighted that a survey distributed in 2011 among breast surgeons and medical oncologists of Uruguay by Valle & Acevedo reported that 67% of respondents had regular tumor boards at their institutions (69% of those working at public centers and 45% of those in private centers).^23^ In the case of Colombia, it was specified that approximately 10% of the centers that provided cancer care were designated as “Centers of Excellence”. In such institutions, a series of indicators are documented to audit the quality of care based on international standards and could have data on MDC implementation. In Mexico, individual clinical researchers and healthcare providers have had the initiative to document MDC uptake, as is the case of Amengol-Alonso et al. that published that 97% of breast cancer patients treated at an institution in Mexico City had been discussed by a multidisciplinary team.^24^ However, no systematic or national measurements are available to objectively quantify MDC in the country.

## Discussion

The management of breast cancer is multi-modal and requires the expertise of a variety of healthcare providers. A multidisciplinary approach represents an opportunity to optimize patient care by enhancing effective communication between team members. However, it has been reported that most clinicians worldwide are unaware of any national or regional guidelines on how interdisciplinary teams should function, nor do they receive any specific training for participating.^19^ Hence, wide variability in the adoption, implementation, and auditing of MDC exists around the globe.^11^^,^^19^^,^^22^ To our knowledge, this is the first study to examine barriers and facilitators for MDC in Latin America.

In Latin America, quality cancer care is limited by fragmented health systems, inadequate distribution of resources, and persistent cultural and geographic barriers.^3^^,^^25^ Given the perceived benefits of MDC, experts from Bolivia, Colombia, Ecuador, Mexico, and Uruguay that participated in this study agreed that the broader implementation of this approach could improve breast cancer care in the region. However, each country had a slightly different opinion on MDC and the team members with an active role in regular interdisciplinary meetings. Succinctly, the experts agreed that MDC was comprised of a team of health care providers who discuss optimal diagnostic and therapeutic strategies on a case-by-case basis, with specialists in medical oncology, breast surgery, radiation oncology, plastic surgery, palliative care, psychology, genetics, and nurses considered essential for discussing patient care. Importantly, the notion that MDC is only possible through a tumor board-like meeting should be challenged and clarified. In our opinion, the vital aspect of this discussion is the effective interaction of the different specialists at each step of the care process.

This study found significant variability in MDC adoption, both at a country level and depending on whether care was provided at private or public health institutions. It was estimated that a multidisciplinary discussion occurs in less than 90% of cases in all countries, occurring in as little as 10% in public settings of Ecuador. Hence, it is evident that a significant proportion of breast cancer patients from Latin America have yet to benefit from the attention and care of a multidisciplinary team. In contrast, the minimum standard of care established by the European Society of Breast Cancer Specialists (EUSOMA) requires that at least 90% of patients are discussed by a multidisciplinary team (target >99%) to ensure optimal patient outcomes.^26^ According to the EP, this goal is achieved only in Montevideo, Uruguay, and some academic centers in Mexico. However, the proportion of patients that receive MDC in each country according to the EP should be interpreted with caution as it is possible that the proportion of cases discussed in MTB at a regional level were overestimated by the panelists.

The EP highlighted a series of barriers limiting the optimal execution of MDC, both in public and private care systems. At a system level, insufficient resources and inadequate infrastructure were cited as prevalent impediments to MDC. At a physician level, low motivation to participate, excessive workload, and inadequate leadership were identified. At a patient level, seeking healthcare at multiple centers could contribute to fragmented services and a lack of coordination between the different specialties. Different strategies to broaden MDC implementation were suggested and involved governmental entities, medical societies, individual institutions, healthcare professionals, and patients. Importantly, effective implementation of this model of care will require the active involvement of all stakeholders and the development of regional context-dependent solutions.

Based on the EP discussion, efforts should be made to quantify the cost-effectiveness of MDC meetings at an institutional level. In the absence of objective and precise measurements of the impact of multidisciplinary breast cancer care, government agencies may not consider the need to prioritize MDC. Research and dissemination of results of individual MDC initiatives can provide valuable evidence for policymakers to promote its use at a regional level. Furthermore, driven institutions should invest in required equipment to conduct interdisciplinary discussions (e.g., a designated meeting room free of distractions, teleconferencing software, and integrated medical records), establish clear protocols for MDC (including frequency and duration of meetings, professionals that should participate, and importantly, documentation of the management strategies discussed), guarantee adequate remuneration to healthcare providers participating in MDC meetings, and appoint a team leader who can ensure the active participation and collaboration of every team member irrespective of existing professional hierarchies. On the other hand, healthcare professionals should have a protected time for participating in interdisciplinary meetings to increase attendance and promote the availability of all the information required for adequate case discussion when presenting cases at an MDC meeting. Ultimately, all involved in MDC should recognize that a functional and effective meeting allows for comprehensive and evidence-based decisions to be made, with a direct positive impact on patient outcomes.^9^^,^^27^^,^^28^

Other strategies to promote MDC in resource-constrained settings include instituting mini-tumor boards when specialists of all areas of cancer care are not available.^29^ This could be useful, for example, in public centers of Bolivia where the absence of geneticists was cited as a barrier for MDC. Similarly, the partnership of national referral centers and international collaborators with centers that do not have adequate access to MDC can tackle geographic disparities. Other disparities may be due to technological development within each country and within each institution and the ability of each hospital to encourage and access meetings that are available to more specialists through virtual resources.

Such an approach has had successful results, according to some of the participating experts. Additionally, technological advances should be used to facilitate MDC. The feasibility and practical aspects of having virtual interdisciplinary meetings are some of the lessons learned with the COVID-19 pandemic.^30^ There will likely be delays in diagnosing and treating breast cancer after the COVID-19 pandemic. However, taking advantage of the growth and development of telemedicine and teleconferences will allow strengthening multidisciplinary care and mitigating the clinical challenges presented after the COVID-19 pandemic.

The concept of the need for an in-person meeting to allow for MDC has proven obsolete and should be abandoned. Even after social-distancing measures relax, most healthcare providers expect MDC meetings to continue in the format of teleconferences or become hybrid in-person & virtual events.^22^ Telemedicine interdisciplinary meetings represent a valuable opportunity to attain high-level expertise in centers with poor access to specialist care. It is also an opportunity to establish international twinning programs between centers in high-, medium-, and low-income countries where knowledge, expertise, and experiences can be disseminated.^31^^,^^32^

Examples of successful international initiatives to promote MDC include mandated interdisciplinary team meetings to obtain center accreditation and clear regional guidelines on how MDT should be undertaken. The first is exemplified by the American College of Surgeons that, for providing Commission on Cancer (CoC) accreditation in the United States, a minimum of 15% of the annual analytic caseload must be discussed at multidisciplinary cancer case conferences.^33^ The second is illustrated by the report “The Characteristics of an Effective Multidisciplinary Team (MDT)” published by the National Cancer Action Team in the United Kingdom, which establishes clear guidance on the team members that should participate in MDC, responsibilities of those in leadership roles, the necessary infrastructure for meetings, and organizational aspects of successful programs.^34^ The development of similar initiatives in Latin America could prove beneficial. Ultimately, local leaders are essential to promote initiatives that will foster MDC adoption at a regional level. Motivated healthcare providers should endorse MDC standardization, demand the optimization of working conditions, sensitize leaders about the need for this strategy, and educate patients and healthcare workers alike on the benefits of regular MTB.

The selection method based on convenience and ease of access to clinicians who participated during the panel of experts can be considered a limitation of this study. Not including all Latin American countries, only five that showed interest in participating also constitute a limitation. Additionally, other aspects of multidisciplinary care have possibly not been incorporated and have not been discussed and analyzed. Likewise, the information collected has a subjective component as it depends on the experience of professionals in their countries and their different workplaces. Therefore, each aspect discussed in the panel of experts could be the subject of local research in each country independently.

## Conclusion

The increasing complexity of breast cancer management prevents a single specialty from covering all the needs of a given patient. Unlike the classical "one-size-fits-all" approach, the provision of personalized care requires a multidisciplinary approach. However, MDC is not uniformly available in Latin American countries at present. In the five countries included in this study, telemedicine initiatives, international collaborations, and increased uptake of electronic medical records are key tools to facilitate MDC in the modern era. Ultimately, the provision of comprehensive, high-quality MDC to improve patient outcomes is an achievable goal that depends on the motivation of individual healthcare providers to propose locally adapted solutions and promote this practice at a regional level.

## Contributors

CB: Conceptualization, funding acquisition, investigation, supervision, validation, visualization, writing – original draft, writing – review & editing

GSV: Conceptualization, data curation, formal analysis, funding acquisition, investigation, methodology, software, supervision, visualization, writing – original draft, writing – review & editing

CVG: Conceptualization, investigation, validation, visualization, writing – review & editing

AO: Conceptualization, investigation, validation, writing – review & editing

MAL: Conceptualization, investigation, validation, writing – review & editing

AMB: Data curation, formal analysis, investigation, methodology, software, visualization, writing – original draft, writing – review & editing

ASF: Investigation, validation, visualization, writing – review & editing

CAC: Conceptualization, funding acquisition, investigation, methodology, project administration, resources, writing – review & editing

All authors had full access to all the data in the study and accept responsibility to submit for publication.

## Data sharing statement

All de-identified data will be available in its original language by contacting the corresponding author.

## Declaration of interests

Productos Roche S.A funded the research group activities, including design, data collection, and analysis, but they did not intervene and stay sidelines of the carried-out activities.

CB reports grants from: Pfizer, Pharma Mar, Polyphor, Henlius Biotech, Shanghai, Merck KGaA, Millennium, LEO Pharma, ImClone Systems, Exelixis, Medivation, Asana Biosciences, AB Science, Abraxis Biosciences, Daiichi Sankyo, Bristol-Myers Squibb, BioMarin, Astellas Pharma, AbbVie, Merck (MSD), Merrimack, Mylan, Taiho Pharmaceutical, Sanofi, GlaxoSmithKline, Roche/Genentech, Lilly, Boehringer Ingelheim, Novartis, AstraZeneca, Amgen, Pfizer. Personal fees from Boehringer-Ingelheim, Sanofi, Lilly, Zodiac, Astra Zeneca, MSD, Bayer, Eisai, Roche/Genentech, Pfizer, Novartis, GSK. Stocks from: MedSIR, Biomarker, Tummi.

CVG reports grants from: Productos ROCHE SA, AstraZeneca, grants and personal fees from Roche, personal fees from Novartis, Pfizer, Lilly, Myriad Genetics.

GSV, AO, MAL, AMB, ASF, CAC: None to declare.

## Appendix Supplementary materials

Image, application 1
