# Supplementary material for: Barriers and facilitators to provide multidisciplinary care for breast cancer patients in five Latin American countries: A descriptive-interpretative qualitative study
Source: Lancet Reg Health Am. 2022 Apr 7;11:100254. doi: 10.1016/j.lana.2022.100254 (PMC9904076; doi:10.1016/j.lana.2022.100254)
Supplement: Supplementary file 1 [file mmc1.docx]

**SUPPLEMENTARY MATERIAL**

TABLE OF CONTENTS

[Supplementary Material 1. Teams and members 1](#_Toc88928765)

[Supplementary Material 2. Guiding questions 3](#_Toc88928766)

[Supplementary Material 3. Definition of multidisciplinary care and the proposed team 4](#_Toc88928767)

[Supplementary Material 4. Percentage of access to MDC in public and private contexts 5](#_Toc88928768)

# Supplementary Material 1. Teams and members

| **Developer Group-Clinical Experts** | |
| --- | --- |
| **Name:** | **Profile** |
| Carlos Barrios | Medical oncologist |
| Cynthia Villareal Garza | Medical oncologist |
| Andrés Ossa | Oncologist / Breast Surgeon |
| Milton Lombana | Medical oncologist |
| Carlos Alberto Castro | Epidemiologist |
| Guillermo Sánchez-Vanegas | Epidemiologist, Public Health Specialist |
| Angélica Monterrosa-Blanco | Epidemiologist |
| **Expert Selection -Mexico** | |
| **Name** | **Profile** |
| Eva Ruvalcaba Limón | Surgical Oncologist |
| Georgina Garnica | Medical oncologist |
| Cesar Lara | Pathologist |
| **Expert Selection - Colombia** | |
| **Name** | **Profile** |
| Andrés Ossa | Oncologist / Mastologist Surgeon |
| Milton Lombana | Medical oncologist |
| Andrés Yepes | Medical Oncologist |
| Johanna Ibarra | Mastologist |
| Fernanda Sua Light | Pathologist |
| Sabrina Herrera | Pathologist |
| Roger Henao | Radiation Oncologist |
| Jose Alejandro Esguerra | Radiation Oncologist |
| **Expert Selection - Ecuador** | |
| **Name** | **Profile** |
| Luis Unda | Medical oncologist |
| Paola Merchán | Medical oncologist |
| **Expert Selection - Bolivia** | |
| **Name** | **Profile** |
| Dr. Lucia Richter | Medical oncologist |
| Niño de Guzmán | Oncologist / Mastologist Surgeon |
| Jose Luis Gonzalez | Pathologist |
| Carolina Hemestrosa | Pathologist |
| Ligia Avilés | Radiologist |
| **Expert Selection - Uruguay** | |
| **Name** | **Profile** |
| Miguel Dalcín | Pathologist |
| Virginia Ortega | Pathologist |
| Carlos Acevedo | Surgeon |
| Guillermo Laviña | Surgeon |
| Veronica Terzieff | Medical oncologist |
| Franco Xavier | Medical oncologist |
| Mauricio Luongo | Radiologist |
| Álvaro Notejane | Radiologist |

# Supplementary Material 2. Guiding questions

- **QUESTION 1.** What is the definition of multidisciplinary care for the diagnosis and management of breast cancer patients?  **QUESTION 2.** Who makes up a multidisciplinary care team for the diagnosis and treatment of breast cancer?
- **QUESTION 3.** In the private sector, what is the percentage of breast cancer patients that have access to multidisciplinary care?
- **QUESTION 4**. In the public sector, what is the percentage of breast cancer patients that have access to multidisciplinary care
- **QUESTION 5.** In the private sector, what are the five main barriers for the implementation of multidisciplinary care for breast cancer patients?
- **QUESTION 6.** In the public sector, what are the five main barriers for the implementation of multidisciplinary care for breast cancer patients?
- **QUESTION 7.** Describe five effective strategies that would help to implement multidisciplinary care in breast cancer patients.
- **QUESTION 8.** According to the experience of your team, describe two effective strategies that have worked for the implementation of multidisciplinary care.
- **QUESTION 9.** According to the experience of your team, has the impact of multidisciplinary care for breast cancer patients been measured? If so, how was it measured?

**Supplementary Material 3. Definition of multidisciplinary care and the proposed team**

| **Country** | **Definition of MDC** | **MDC team** |
| --- | --- | --- |
| **Bolivia** | Combination of scientific knowledge and expertise in specific areas of breast cancer management with the sole objective of providing personalized care that is planned according to the best diagnostic, treatment, and follow-up strategies available for this disease. | For patient diagnosis: radiology, pathology, and nuclear medicine  For treatment decisions: breast surgery, clinical oncology, surgical oncology, radiation oncology, psycho-oncology, palliative care, plastic surgery, nutrition, physical therapy, social services, genetics, and gynecology |
| **Colombia** | Quality patient-centered care guided by breast cancer specialists of who can determine the optimal management strategies and treatment sequences for each patient based on clinical guidelines, international standards, and institutional protocols. | Core group: clinical oncology, breast surgery, radiation oncology, pathology, radiology, rehabilitation, palliative care, psychology, nurse navigator / nurse oncologist, social services, genetics, plastic surgery  Group Leader: breast surgery or clinical oncology |
| **Ecuador** | Personalized patient care that is based on a case-by-case analysis by a team comprised by multiple specialties involved in the management of breast neoplasms. Through regular meetings, the team can discuss diagnostic and therapeutic options and arrive at an optimal treatment strategy based on available evidence. This process improves communication and coordination between differ healthcare providers involved in patient care and enhances the management decisions. | Oncological surgery / breast surgery, gynecology, medical oncology, radiation oncology, radiology, interventional radiology, pathology, nuclear medicine, molecular medicine, genetics, nursing, psychology, social services, palliative care. |
| **Mexico** | Patient-centered care provided by a team of experts in breast cancer management that offers accurate diagnosis and optimal treatment strategies. | Maxillofacial surgery, odontology, oncological surgery, gynecology, medical oncology, radiation oncology, radiology, physical therapy, palliative care, plastic surgery, pathology, genetics, psycho-oncology, nutrition |
| **Uruguay** | A process where experts from several specialties collaborate to make decisions about patient care. | Core team: medical oncology, surgery, radiotherapy, pathology, nursing  Variable team: internal medicine, genetics, physical therapy, plastic surgery, psychology, social services, cytology. |

# Supplementary Material 4. Percentage of access to MDC in public and private contexts

| **Country** | **MDC Access – Public institutions** | **MDC Access - Private institutions** |
| --- | --- | --- |
| **Bolivia** | Only 25-35% of patients are discussed by multidisciplinary teams as this is not covered by public healthcare services. | 50-70% of patients receive MDC. Overall, low availability of clinical geneticists’ complicate access to MDC. |
| **Colombia** | Approximately 40% of patients can receive MDC in public institutions. | It is estimated that 70% of patients receive MDC. |
| **Ecuador** | 80-90% receive MDC. | 10-20% receive MDC. |
| **Mexico** | In university hospitals, close to 90% are discussed in interdisciplinary teams. In general, all patients treated at public institutions have access to the specialists necessary for quality care. However, all services might not be available at a single center, which complicates the assessment of the proportion of patients that ultimately benefit from multidisciplinary discussion and care. | If this care is provided in an academic hospital, more than 50% of patients have access to multidisciplinary teams. Even close to 90%, according to a study carried out with 13 hospitals. However, individual practices are highly heterogeneous, and no objective data is available to provide an accurate estimate. |
| **Uruguay** | Approximately 64% of patients receive MDC, although it varies by region. It is estimated that all patients in Montevideo who public medical care receive MDC. | According to the panelists, 48% of patients receive MDC. |
